# The effect of pH and ionic strength on the adsorption of glyphosate onto ferrihydrite

**DOI:** 10.1186/s12932-019-0063-1

**Published:** 2019-05-24

**Authors:** Rodrigo C. Pereira, Pedro R. Anizelli, Eduardo Di Mauro, Daniel F. Valezi, Antonio Carlos S. da Costa, Cássia Thaïs B. V. Zaia, Dimas A. M. Zaia

**Affiliations:** 1 Laboratório de Química Prebiótica, Departamento de Química-CCE, Universidade Estadual de Londrina, Londrina, Brazil; 2 Departamento de Física-CCE, Universidade Estadual de Londrina, Londrina, Brazil; 3 Departamento de Agronomia-CCA, Universidade Estadual de Maringá, Maringá, PR 87020-900 Brazil; 4 Departamento de Ciências Fisiológicas-CCB, Universidade Estadual de Londrina, Londrina, PR 86051-990 Brazil

**Keywords:** Glyphosate, Adsorption, Iron oxide, Ferrihydrite

## Abstract

Although, glyphosate (*N*-(phosphonomethyl) glycine) is one of the most widely used herbicides in the world, its interaction with poorly crystalline iron oxides, such as ferrihydrite, is not well studied. In this research, we examined the adsorption of glyphosate onto ferrihydrite using infrared spectroscopy (FT-IR), electron paramagnetic resonance spectroscopy (EPR), adsorption kinetic models and adsorption isotherm models. The effect of pH and sodium chloride concentration on the adsorption of glyphosate onto ferrihydrite as well as the effect of extractors (CaCl_2_ 0.010 mol L^−1^ and Mehlich) on the desorption of glyphosate were also evaluated. There are two important findings described in this work. First, 84% of adsorbed glyphosate strongly interacted to ferrihydrite as an inner-sphere complex and phosphate and amine groups are involved in this interaction. Second, an increase of sodium chloride salt concentration increased the adsorption of glyphosate onto ferrihydrite. The non-linear Langmuir model and pseudo second order model showed a good agreement of theoretical limit of glyphosate adsorbed onto ferrihydrite, 54.88 µg mg^−1^ and 48.8 µg mg^−1^, respectively. The adsorption of glyphosate onto ferrihydrite decreased when the pH increased. Under the conditions used in this work, EPR spectra did not show dissolution of ferrihydrite. Surface area, pore volume and pH_pzc_ of ferrihydrite decreased after adsorption of glyphosate.

**Electronic supplementary material:**

The online version of this article (10.1186/s12932-019-0063-1) contains supplementary material, which is available to authorized users.

## Introduction

Herbicides are classified as micropolluting agents because of their low concentration in the environment. When in contact with the soils, if they do not undergo adsorption and/or degradation, they can move into groundwater or rivers causing multiple kinds of damage to the environment. Decomposition and adsorption of herbicides by soils depend on pH, humidity, cation exchange capacity, temperature and content of metals (transition metals), minerals (iron oxides and aluminum, clay minerals, etc.) and organic matter [1–4]. Thus, the mechanism of adsorption and degradation of each herbicide depends on its physicochemical properties as well as the specific soil properties [2, 4].

Glyphosate [*N*-(phosphonomethyl) glycine-C_3_H_8_NO_5_P] is a non-selective, systemic and post emergent herbicide that was discovered at Monsanto in 1970 by a group of scientists led by Dr. John Franz. It is among the most widely used herbicides in the world [1]. Glyphosate blocks the enzymatic synthesis of aromatic amino acids (phenylalanine, tyrosine, tryptophan) in plants. These amino acids are precursors in the synthesis of lignin, alkaloids, flavonoids, benzoic acids and vitamin K [5, 6]. In soil the principal decomposition product of glyphosate is aminomethylphosphonic acid [1, 2].

Iron is the fourth most abundant element in the Earth’s crust and there are 16 iron-oxygen compounds including oxides, hydroxides, and oxides-hydroxides. Iron oxides are common in nature; they can be found in soils, rocks, lakes, rivers, seafloor sediments, the atmosphere, and living beings. Iron oxides consist of an iron atom bound to O and/or OH [7]. Ferrihydrite was first described by Chukhrov in 1973 [7]. It is a poorly ordered iron oxide that it is usually characterized as being 2-lines or 6-lines because of the number of reflections in the X-ray diffractograms [7]. Despite the difficulty in identifying ferrihydrite in soils, in recent years there has been increased interest in it because its surface area (between 200 and 400 m^2^ g^−1^) is much higher than the other iron oxides (< 100 m^2^ g^−1^) [7, 8]. Consequently, ferrihydrite is an excellent adsorbent of certain organic molecules. Soils formed under humid weather conditions have poorly crystalline minerals such as ferrihydrite [7, 9]. The weathering process of primary and secondary minerals liberates Fe, Al, and Si into the soil solution that are either complexed by the soil organic matter or precipitate as poorly crystalline minerals [7, 10]. Fe-poorly crystalline forms include the minerals ferrihydrite, schwertmannite and green rust or fougerite [7, 9, 10]. In oxidized soils and sediments, ferrihydrite is the preferred mineral formed [7, 9, 11]. These poorly crystalline forms due to their nanosizes have very high specific surface area (ranging from < 100 up to 1000 m^2^ g^−1^) with a large distribution of the ferrol ([]FeOH)) surface functional group that presents pH at the point of zero charge (pH_PZC_) above 7.0, i.e. in the alkaline range [7]. Ferrihydrite present in soils with such high specific surface area and high pH_PZC_ have large values of the positive surface charge with whom the phosphate functional group from glyphosate will form inner sphere complexes [11, 12].

In the literature there are several studies showing the interaction between glyphosate and iron oxides [13–20]; between glyphosate and metals [21–24]; between glyphosate and organic matter [14, 25]; between glyphosate and clay minerals and glyphosate and soils [26–28].

Because of goethite’s (FeO(OH)) high stability, crystallinity and abundance in soils, most of the studies of the interaction between glyphosate and iron oxides were carried out with goethite [13–15, 17–20]. Other iron oxides have also been studied: McConnell and Hossner [13] and Gimsing and Borggaard [16] studied the interaction between glyphosate and hematite (Fe_2_O_3_) and ferrihydrite/hematite, respectively. In general, these studies showed that the iron of iron oxides interacts with glyphosate through a phosphate group. Other groups (amine and carboxylic) could also be involved. According to Dideriksen and Stipp [18], goethite interaction occurs with the hydroxyl of the phosphate groups and carboxylic monodentate and bidentate forms, with the simultaneous interaction of the two groups hindered by a steric effect.

In the literature, there is a great dearth of studies of the interaction of glyphosate with ferrihydrite and because this iron oxide has a large surface area, being an important adsorber of organic micropollutants, more studies of it are needed.

In the present work we studied, using infrared and electron paramagnetic resonance spectroscopies, the adsorption (isotherm) and desorption (CaCl_2_ 0.010 mol L^−1^ and Mehlich extractors) of glyphosate on ferrihydrite and the interaction between glyphosate and ferrihydrite. The effect of sodium chloride concentration and pH on the adsorption of glyphosate onto ferrihydrite, the pH of the point of zero charge (pH_pzc_), the kinetics of adsorption of glyphosate on ferrihydrite and its surface area were also evaluated.

## Materials and methods

### Materials

All reagents used were analytical grade.

#### Synthesis of ferrihydrite

**Fig. 1:**
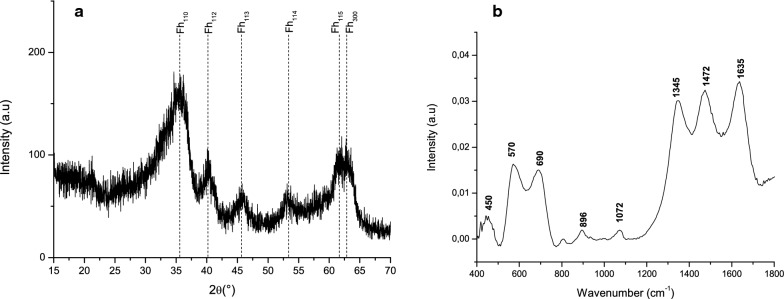
**a** X-rays diffractogram of ferrihydrite and **b** infrared spectrum of ferrihydrite

The 6-line ferrihydrite was prepared according to Cornell and Schwertmann [28]. All synthesis was carried out at 75 °C in a plastic box. Briefly, about 66 mL of a KOH (1.0 mol L^−1^) solution was added into 100 mL of Fe(NO_3_)_3_.9H_2_O (0.20 mol L^−1^) solution with constant stirring. The addition of the potassium hydroxide was performed slowly along 1 h until the pH was 7.5. The precipitated material was filtered on a vacuum system and washed 6 times with ultrapure water, in order to removing nitrate excess. The material was lyophilized and the 6-line ferrihydrite was characterized by X-ray diffraction and FTIR (Fig. 1).

### Methods

#### Adsorption isotherm

About 60 mg of ferrihydrite was placed in conical tube (15 mL) and after it was added 10 ml of following glyphosate solutions 0.6, 1.2, 1.8, 2.4, 3.0, 3.5, 4.1, 4.7, 5.3 and 5.9 mmol L^−1^. Each isotherm was performed in quadruplicate. Glyphosate was dissolved in sodium chloride solutions (0.010 and 0.10 mol L^−1^). The pH of the solutions was adjusted to 5.00 with sodium hydroxide or hydrochloric acid 0.10 mol L^−1^. The suspensions were stirred for 24 h at 30 °C. After, pH was measured and samples were centrifuged at 9000 rpm for 10 min. The supernatant was used for the glyphosate determination [29].

The results of glyphosate adsorption onto ferrihydrite were fitted to non-linear isotherm models (Langmuir and Freundlich), the non-linear model of single-site Langmuir–Freundlich [30, 31] and non-linear Langmuir isotherm model modified by Barrow et al. [32].

Non-linear Langmuir isotherm model

1 $$\uptheta = \varvec{ }\frac{{k_{eq} bC}}{{\left( {1 + C} \right)}}$$

where C (mg L^−1^) is concentration of glyphosate in solution after the equilibrium, θ (mg g^−1^) is the concentration of glyphosate adsorbed onto ferrihydrite (difference between initial glyphosate concentration and the concentration after the equilibrium), b (mg g^−1^) is the theoretical limit of adsorbed glyphosate onto ferrihydrite, keq (L mg^−1^) is equilibrium constant (adsorbate-adsorbent).

Non-linear Freundlich isotherm model

2 $$\uptheta = \text{K}_{\text{f}} \text{C}^{\text{n}}$$

where C (mg L^−1^) is concentration of glyphosate in solution after the equilibrium, θ (mg g^−1^) is the concentration of glyphosate adsorbed onto ferrihydrite (difference between initial glyphosate concentration and the concentration after the equilibrium), K_f_ and n are empirical constants.

Non-linear single-site Langmuir–Freundlich isotherm model

3 $$\uptheta = \varvec{ }\frac{{b\left( {KC} \right)^{n} }}{{1 + \left( {KC} \right)^{n} }}$$

where C (mg L^−1^) is concentration of glyphosate in solution after the equilibrium, θ (mg g^−1^) is the concentration of glyphosate adsorbed onto ferrihydrite (difference between initial glyphosate concentration and the concentration after the equilibrium), b (mg g^−1^) is the theoretical limit of adsorbed glyphosate onto ferrihydrite. K and n are empirical constants.

Non-linear Langmuir isotherm model modified by Barrow et al. [32]

4 $$c = a\;\exp \left( {b\;S} \right)S/\left( {S_{\text{max} } - S} \right)$$

where *c* is the observed concentration; *S* is the observed adsorption and *S*_max_ is the maximum adsorption, *a* is a parameter defined as

5 $$a = 1/k_{i} \alpha \;exp\left( { - z_{i} F\psi_{a} /RT} \right)$$

and *b* is a parameter defined as

6 $$b = - z_{i} Fm/RT$$

where *K*_*i*_ is a binding constant for the ion *i* with valency *z*_*i*_, *α* is its proportion present in solution, *F* is the Faraday constant, *ψ*_*a*_ is the mean electric potential experienced by the adsorbed ion, *R* is the gas constant, *T* is the temperature (kelvin) and *m* is defined by

7 $$\psi = \psi_{0} - mS$$

where ψ_0_ is the electric potential in the absence of adsorption.

#### Glyphosate quantification

In 10 mL test tubes was transferred 500 μL of glyphosate solutions, ranging from 60 to 360 µmol L^−1^, 500 μL of ninhydrin solution 5% w/v (weight/volume), 500 μL of sodium molybdate 5% w/v and 5 mL of ultrapure water. The calibration curves were made in triplicate, after mixtures were kept in boiling water bath for 5 min. The solution containing the product, resulting from the reaction of Ruhemann Purple, was cooled to room temperature. The absorbance of the samples was measured at 570 nm [29].

#### Effect of pH and sodium chloride concentrations on adsorption of glyphosate onto ferrihydrite

Glyphosate was dissolved in sodium chloride solutions (0; 0.010 and 0.10 mol L^−1^) at final concentration of 4.4 mmol L^−1^. After, in conical tube (15 mL) containing 30 mg of ferrihydrite, 10 mL of each solution was added to three different sets with four samples each. The pH was adjusted to 2.0, 3.0, 4.0,5.0, 6.0 or 7.0 by addition of HCl or NaOH 0.10 mol L^−1^. The tubes were stirred for 24 h at 30 °C and then centrifuged for 15 min at 9000 rpm. The aqueous phase was used for quantification of glyphosate [29] and the solid was lyophilized and stored for further characterization.

#### Glyphosate desorption

Desorption experiments of glyphosate from ferrihydrite used samples in which adsorption was performed at pH 5.0 and 4.7 mmol L^−1^ of glyphosate. For desorption experiments CaCl_2_ 0.010 mol L^−1^ and Mehlich-1 extractor were used [33, 34]. Mehlich-1 is a mixture of HCl (0.050 mol L^−1^) plus H_2_SO_4_ (0.025 mol L^−1^) [33, 34].

All desorption experiments were carried out sequential three times using 2.0 mL of extractor (CaCl_2_ or Mehlich). The samples were stirred for 24 h at 30 °C, after centrifuged for 15 min at 9000 rpm. The supernatant of each desorption was separated and stored in polyethylene bottles until readings were taken. The glyphosate determinations were carried out using supernatant of each desorption of extracts by spectrophotometric method with ninhydrin reagent [29].

#### pH at the point of zero charge (pH_pzc_)

pHpzc was performed with three repetitions. For each repetition, 50 mg of ferrihydrite were added in four Eppendorf tubes. In two tubes, 125 µL of KCl solution (1.0 mol L^−1^) and other two 125 µL of ultra-pure water were added. All tubes were stirred for 30 min and after 24 h the pH was measured. To calculate the value of pH_pzc_ the Eq. 8 was used [35]:

8 $$pH_{pzc} = 2.pH_{{KCl\left( {1,0 \, \text{mol L}^{ - 1} } \right)}} - pH_{ultrapure \, water}$$

#### Adsorption kinetics of glyphosate onto ferrihydrite

Glyphosate kinetics adsorption process onto ferrihydrite was performed with 60 mg of ferrihydrite in conical tube (15 mL) and 10 ml of glyphosate solutions (3.0 mmol L^−1^). Glyphosate was dissolved in sodium chloride (0.1 mol L^−1^) and the pH was adjusted to 5.0 with sodium hydroxide and hydrochloric acid (0.10 mol L^−1^). The suspensions were stirred for 15, 30, 45, 60, 90, 120 and 180 min, at 307.6 K. After each run, pH was checked and samples were centrifuged at 9000 rpm for 15 min. All experiments were performed in triplicate. The supernatant was used for determination of glyphosate [29].

The pseudo-first-order model is described by the equation [36]:

9 $$\log \left( {q_{e} - q_{t} } \right) = \log q_{e} - \frac{{k_{1} }}{2.303}t$$

where *k*_1_ (min^−1^) is the pseudo-first-order constant, qe is the amount of glyphosate adsorbed (mg g^−1^) at the equilibrium concentration and qt is the amount adsorbed (mg g^−1^) at time t.

The pseudo-second-order model is given by the following equation [36]:

10 $$\frac{1}{{q_{t} }} = \frac{1}{{k_{2} q_{e}^{2} }} + \frac{1}{{q_{e} }}t$$

where *k*_2_ (g mg^−1^ min^−1^) is the pseudo-second-order constant, q_e_ is the amount of glyphosate adsorbed (mg g^−1^) at the equilibrium concentration and q_t_ is the amount adsorbed (mg g^−1^) at time t.

The intra-particle diffusion model is given by the following equation [36]:

11 $$q_{t} = k_{d} t^{1/2} + C$$

where q_t_ is the amount adsorbed (mg g^−1^) at time t, k_d_ is the intra-particle diffusion rate constant (m g^−1^ min^−1/2^) and C is the intercept which is related to the magnitude of the diffusion resistance layer.

#### Fourier transform infrared spectroscopy (FTIR)

Lyophilized ferrihydrite samples after adsorption experiments were analyzed in ATR-FTIR. The spectra were obtained with a resolution of 4 cm^−1^ in range 4000–400 cm^−1^ in Bruker- Vertex 70 spectrometer equipped with ATR accessory with a Ge crystal 45°.

#### Electron paramagnetic resonance spectroscopy (EPR)

Samples of the ferrihydrite were analyzed in EPR spectroscopy using a JEOL spectrometer (JES-PE-3X) at X band (around 9 GHz) with modulation amplitude of 20 G. The magnetic field used was 100 kHz and the analysis were made at room temperature (23 °C). For all analysis, the *g*-marker DPPH (2,2-diphenyl-1-picryl-hydrazyl) was used, and as line intensity level, using its spectral line. The g-factor (g_2_) of each line in the EPR spectra was calculated using the equation:

12 $$g_{2} = \frac{{g_{1} B_{1} }}{{B_{2} }}$$

where g_1_ is the value of the DPPH g-factor (g = 2.004) and B_1_ and B_2_ are the magnetic field values of the central line of DPPH and sample, respectively.

#### Specific surface area analysis

The specific surface area analysis was performed on equipment High Speed Gas Sorption Analyzer Version 11:02. Dollimore and Heal (DH) Barrett–Joyner Halenda (BJH) Brunauer, Emmett and Teller (BET) methods were used for the determination of pore size, volume and surface area, respectively. The samples were pretreated at 120 °C under vacuum for 3 h. The measurements were performed in N_2_ (77.3 K) temperature. The results were analyzed by the software Nova Win 11.0.

#### X-ray diffraction

Ferrihydrite was analyzed by powder X-ray diffraction using a Panalytical X’Pert PRO MPD diffractometer using Cu Kα radiation (40 kV, 30 mA) and an iron filter in a step-scanning mode (0.02°2θ/0.6 s). All peak positions were analyzed using Grams software (Thermo Scientific, version 8.0).

## Results and discussion

### Characterization of ferrihydrite

The X-rays diffractograms of the ferrihydrites were all with 6-lines (Fig. 1a) [7].

The FT-IR spectrum of ferrihydrite (Fig. 1b) shows bands at 1635 cm^−1^, 1472 cm^−1^/1345 cm^−1^/1072 cm^−1^/896 cm^−1^ and 690 cm^−1^/570 cm^−1^/450 cm^−1^, which can be attributed to adsorbed lattice water (CO_3_)^2−^ stretching/bending, and angular deformation of O–H group/Fe–O stretching, respectively [7, 37, 38]. The bands at 1472 cm^−1^/1345 cm^−1^/1072 cm^−1^/896 cm^−1^ appear because CO_2_ from the air was absorbed by the solution and the ferrihydrite adsorbed it as carbonate [37, 38].

### Specific surface area

The specific surface area and pore size distribution of the ferrihydrite are close to values in the literature [7, 39, 40] (Table 1). Adsorption of glyphosate onto ferrihydrite significantly decreases the surface area and pore volume (Table 1). However, the decrease in surface area and pore volume could be not attributed solely to glyphosate because Na^+^ and Cl^−^ were also adsorbed by the ferrihydrite. Vieira et al. [41] observed a decrease in the surface area of ferrihydrite and magnetite after adsorption of cysteine.

**Table 1:** Results of the adsorption/desorption of N_2_ at 77 K in ferrihydrite and effect of sodium chloride concentration and initial pH values on the pH at the point of zero charge (pH_PZC_)

Applied method	Ferrihydrite^a^	Ferrihydrite + glyphosate^b^	
BET surface area (m^2^ g^−1^)	234	196	
BJH pore volume (cm^3^ g^−1^)	0.116	0.091	
DH pore volume (cm^3^ g^−1^)	0.115	0.091	
BJH pore size (nm)^a^	1.69	1.68	
DH pore size (nm)^a^	1.69	1.68	

[NaCl]/mol L^−1^	pH_PZC_		
	Glyphosate/µmol		
	0.0	0.0	41.4
	Without pH adjust	pH = 5.0	
0	8.47	7.76	4.96
0.01	8.20	7.08	5.10
0.1	8.33	8.52	4.99

*BET* Brunauer–Emmett–Teller, *BJH* Barrett–Joyner–Halenda, *DH* Dollimore-Heal

^a^ Pore radius, ^b^ ferrihydrite used as synthesized, ^c^ ferrihydrite plus 30.0 µmol of glyphosate dissolved in 10 mL of 0.10 mol L^−1^ of NaCl at pH = 5.0. For the experiments of pH_PZC_ was used an initial pH of 5.0. For all experiments was used 60 mg of ferrihydrite. The results were a mean of two experiments

### pH at the point of zero charge-pHpzc

An important characteristic of materials is the pH at the point of zero charge (pH_PZC_). When the pH is above the pH_PZC_ the material is negatively charged and below this value the material is positively charged [42].

The pH_PZC_ value of this synthetic ferrihydrite (without further preparation) was 8.47. After the ferrihydrite was mixed with sodium chloride solutions (0.010 and 0.10 mol L^−1^) a small decrease in pH_PZC_ was observed (Table 1)—probably due to a small adsorption of chloride. However, when the pH was adjusted to 5.0 with a HCl solution, a large decrease of the pH_PZC_ was observed for the sample without sodium chloride and the sample with 0.010 mol L^−1^ of sodium chloride (Table 1). This decrease in pH_PZC_, suggests that Cl^−^ ions are adsorbed onto the positively charged sites of the ferrihydrite, causing changes in surface charges. The adsorption results in a higher number of negatively charged sites, which results in the decrease of pH_PZC_. The Cl^−^ adsorption may occur on these samples because there is an excess of these ions due to the addition of HCl to adjust the pH. Although a large decrease in pH_PZC_ occurred for a 0.010 mol L^−1^ of sodium chloride solution, the Na^+^ concentration of this solution did not change. Rundberg et al. [43] did not observe Na^+^ adsorption onto goethite at pH < pH_PZC_.

Conversely, at high sodium chloride concentration (0.10 mol L^−1^) a decrease in the pH_PZC_ was not observed (Table 1). Analysis of Na^+^ and Cl^−^ ions after being in contact with ferrihydrite for 24 h showed that approximately 8-10% of these ions from the original solution of sodium chloride (0.10 mol L^−1^) were adsorbed onto ferrihydrite. Although ion pairs are usually formed between two polyvalent ions or between one monovalent ion and other polyvalent ion [44], the formation of ion pairs between two monovalent ions can occur [45, 46]. According to Johnson and Pytkowicz [45], at 0.7 mol L^−1^ of NaCl approximately 0.112 mol L^−1^ is present as NaCl ion pairs. Computer simulations performed by Degrève and da Silva [46] also showed that for 1.0 mol L^−1^ of NaCl approximately 0.109 mol L^−1^ are present as NaCl ion pairs. Thus, at high sodium chloride concentration, Na^+^ and Cl^−^were adsorbed onto ferrihydrite as an ion pair and so the pH_pzc_ did not change (Table 1).

A large decrease in pH_PZC_ was observed after adsorption of glyphosate onto ferrihydrite (Table 1). Although the conditions of adsorption were different (0.0, 0.01and 0.10 mol L^−1^ of NaCl), the values of pH_PZC_ were very similar to each other (Table 1). In solutions with pH = 5.0, glyphosate has a negative charge at the phosphate group, another at the carboxyl and a positive charge at the amine group (Additional file 1: Figure S1). Since the pH of the solutions was approximately 5.0, a large portion of the glyphosate has two negative charges on the phosphate group (Additional file 1: Figure S1). The interaction between glyphosate and ferrihydrite occurs through phosphate and amine groups. Thus, glyphosate adsorption increases the number of negative charges on ferrihydrite causing the decrease in pH_PZC_ (Table 1). Other authors have observed similar interactions of glyphosate with metals contained in minerals [19, 27, 28].

### Electron paramagnetic resonance spectroscopy-EPR

The EPR spectra of samples of unreacted ferrihydrite and two ferrihydrites after adsorption of glyphosate (NaCl, 0.010 and 0.10 mol L^−1^) are similar with a single strong signal at g = 2.16 (Additional file 2: Figure S2) that is due to hydroxides and oxides of Fe^3+^ [47]. Vieira et al. [41] did not observe changes in the EPR spectra of ferrihydrite that had been in contact with artificial seawater and artificial seawater plus cysteine for 24 h. EPR spectra of the supernatant of glyphosate solutions after contact with ferrihydrite for 24 h did not show a signal for Fe^3+^. The EPR equipment used in these experiments has a limit of detection of 1.0 ppm for Fe^3+^: this means that less than 0.02% of the ferrihydrite could be dissolved by glyphosate. This shows that, under the experimental conditions used in the present work, ferrihydrite was stable.

### Effect of pH and sodium chloride concentration

**Fig. 2:**
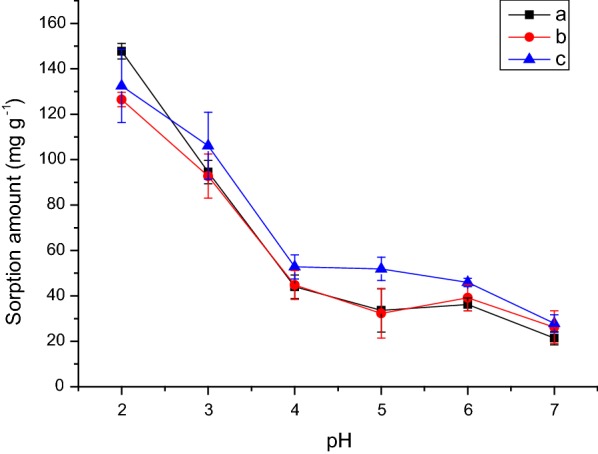
Effect of pH on the adsorption of glyphosate (GPS) onto ferrihydrite (**a**) without NaCl, (**b**) with 0.01 mol L^−1^ of NaCl and (**c**) with 0.10 mol L^−1^ of NaCl

The adsorption of glyphosate onto ferrihydrite decreased when the pH increased (Fig. 2). Glyphosate has, at pH 2.0 to 5.6, one deprotonated oxygen in the phosphate group and above pH 5.6 has two deprotonated oxygens in the phosphate group (Additional file 1: Figure S1). Thus, as the pH of the samples increased, both the surface of ferrihydrite and the glyphosate molecule (Additional file 1: Figure S1) became more negatively charged, decreasing the interaction between them (Fig. 2). This suggests that the interaction that occurs between the glyphosate and ferrihydrite could be electrostatic. Also, McConnell and Hossner [13] and Barja and Afonso/Orcelli et al. [19, 20] observed that adsorption of glyphosate onto clays/hematite/goethite and goethite decreased with an increase in the pH. An inverse trend was observed for cation adsorption onto ferrihydrite, which demonstrates the occurrence of an electrostatic interaction between iron oxides and various adsorbates [39, 48].

The highest adsorption of glyphosate onto ferrihydrite occurred at pH 2.0 without sodium chloride (Fig. 2). However, for the other pH values, an increase in the adsorption of glyphosate onto ferrihydrite was noted with an increase of sodium chloride concentration (Fig. 2). With increasing ionic strength the double layer thickness decreases enhancing the formation of complexes. At pH 7.0 there is no significant difference on the adsorption (p > 0.05). According to Bolan et al. [49], for phosphate, there is a pH (pH point of zero salt effect—pH_PZSE_) for which there is no effect of electrolyte concentration on adsorption: above the pH_PZSE_ the adsorption increases with increased electrolyte concentration and below the pH_PZSE_ the reverse trend occurs. Assuming that glyphosate adsorption has a similar behavior to phosphate adsorption, the pH_PZSE_ can explain the increase of glyphosate adsorption onto ferrihydrite at higher sodium chloride concentration at these pH values. For a material with the same reactive surface, the numeral values of pH_PZC_ and pH_PZSE_ are the same [48]. Gimsing and Borggaard [15] studied the effect of KCl on the adsorption of glyphosate and phosphate onto goethite and observed that there is a higher phosphate adsorption at a higher concentration of KCl at pH 7.0. In the adsorption of glyphosate, the same behavior was observed, but the effect of ionic strength was small compared to the effect on phosphate adsorption. According to the authors, at pH values above 4.5 this behavior is expected for phosphate adsorption. Our results show that glyphosate is adsorbed onto ferrihydrite in a way very similar to that of phosphate because a large increase of adsorption onto ferrihydrite occurred as the NaCl concentration increased (Fig. 2). Thus, even in soils with high concentration of salts, glyphosate will be adsorbed by ferrihydrite and so might cause no damage to the environment. However, the adsorption of glyphosate onto goethite decreased when NaCl concentration increased [20].

### Adsorption isotherms

**Fig. 3:**
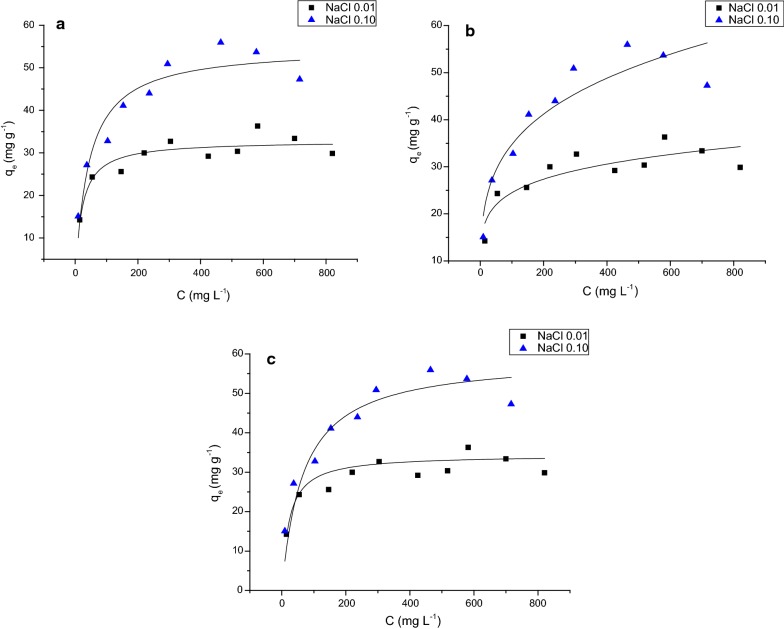
Illustration of the fit of the **a** non-linear Langmuir model, **b** non-linear Freundlich model and **c** non-linear Langmuir–Freundlich model to the data for adsorption of glyphosate onto ferrihydrite with NaCl 0.01 mol L^−1^ and 0.10 mol L^−1^

The results of glyphosate adsorption onto ferrihydrite at pH 5.0 and different NaCl concentrations (0.01 and 0.1 mol L^−1^) were fit to a non-linear Langmuir and Freundlich models and to a non-linear Langmuir–Freundlich model (Table 2; Fig. 3). The nonlinear fit was used for all isotherm models, because according to Kinniburgh [50], Longhinotti et al. [51], Ho [52] and Kumar [53] these fits produced more reliable results of isotherm parameters. The main concern about linear fits that for example Langmuir isotherm model can be linearized in four different types and each one can result in different parameters [51, 52].

**Table 2:** Parameters of non-linear Langmuir, Freundlich and Langmuir–Freundlich for samples of glyphosate adsorbed onto ferrihydrite

[NaCl] mol L^−1^	Model	K	1/n	b	R^2^
0.10	nl-Lang	0.023	–	54.88	0.903
	nl-Freu	11.25	0.245	–	0.881
	nl-Lang-Freu	0.015	0.657	64.83	0.928
0.01	nl-Lang	0.049	–	32.83	0.857
	nl-Freu	11.75	0.160	–	0.780
	nl-Lang-Freu	0.046	0.785	34.40	0.864

Each result was mean of four experiments. The solutions were stirred for 24 h at room temperature, at pH 5.0 with 60 mg of ferrihydrite. nl-Lang: non-linear Langmuir; nl-Freu: non-linear Freundlich; nl-Lang-Freu: non-linear Langmuir–Freundlich single site. K (Langmuir) (L mg^−1^) and K (Freundlich) (mg g^−1^) (L g^−1^): adsorbate–adsorbent affinities; b: maximum adsorption capacity (mg g^−1^); 1/n: empiric Freundlich constant

A pH of 5.0 was chosen since this is a common value found in Brazilian soils [54]. The Langmuir model suggests a monolayer adsorption and there are no interactions among the adsorbed molecules. Adsorption that follows the Freundlich model assumes a heterogeneous surface due to a diversity of adsorption sites and the nature of the ions as free or hydrolyzed species [30, 31]. The Langmuir–Freundlich model combines both Langmuir and Freundlich equations, it can be assumed that, at low concentrations, the adsorption assumes the Freundlich isotherm. However, at high concentrations, the adsorption follows the Langmuir isotherm [55]. The best fit was obtained for the Langmuir and Langmuir–Freundlich models, which indicates that most adsorption of glyphosate onto ferrihydrite was in unique site (Table 2; Fig. 3). This result was consistent with our desorption study described in the “Desorption of glyphosate” section, that indicated the presence of adsorption only through inner-sphere complexes. In addition, it should be pointed out that several other authors studied the adsorption of glyphosate onto iron oxides using the Langmuir isotherm model (Table 3). However, McConnell & Hossner [13] and Orcelli et al. [20] used the Freundlich isotherm model. Although McConnell and Hossner [13] did not give any reason for using this model instead of the Langmuir isotherm model, the high maximum adsorption capacities obtained in their experiments should be noticed (Table 3). Thus, it is probable that interaction among glyphosate molecules occurred and, consequently, the results did not fit with the Langmuir isotherm model. According to Orcelli et al. [20] their data fit well in Freundlich model because this mineral has heterogeneous adsorption sites.

**Table 3:** Surface area, maximum adsorption capacity and maximum adsorption densities values for iron oxides

Mineral	SA (m^2^ g^−1^)	MAC (mg g^−1^)	MAD (µmol m^−2^)	Adsorption conditions	References
Ferrihydrite 6 line	234	54.88^a^	1.39^a^	NaCl 0.10 mol L^−1^, pH 5.0	This work
Ferrihydrite 6 line	234	32.83^a^	0.83^a^	NaCl 0.01 mol L^−1^, pH 5.0	This work
Ferrihydrite 2 line	343	107^a^	1.85^a^	KCl 0.10 mol L^−1^, pH 7.0	[16]
Hematite	33	14.5^a^	2.61^a^	KCl 0.10 mol L^−1^, pH 7.0	[16]
Hematite	11.1	6.31^b^	3.36^b^	pH 4.5	[13]
Hematite	11.1	7.89^b^	4.21^b^	pH 7.0	[13]
Goethite	50.7	16.3^b^	1.90^b^	pH 4.5	[13]
Goethite	50.7	21.6^b^	2.52^b^	pH 7.0	[13]
Goethite	40	10.1^c^	1.50^c^	KCl 0.010 mol L^−1^, pH 7.0	[15]
Goethite	40	11.8^c^	1.75^c^	KCl 0.10 mol L^−1^, pH 7.0	[15]
Goethite	40	11.8^c^	1.75^c^	Ca(Cl)_2_ 0.01 mol L^−1^, pH 7.0	[15]
Goethite	60	21.3^a^	2.10^a^	NaCl 0.01 mol L^−1^, pH 5.0	[19]
Goethite	60	17.2^a^	1.70^a^	NaCl 0.01 mol L^−1^, pH 7.2	[19]

*SA* surface area, *MAC* maximum adsorption capacity, *MAD* maximum adsorption densities

^a^Langmuir isotherm, ^b^Freundlich isotherm, ^c^ initial concentration 2.5 mL of 80 mmol L^−1^ of glyphosate

**Fig. 4:**
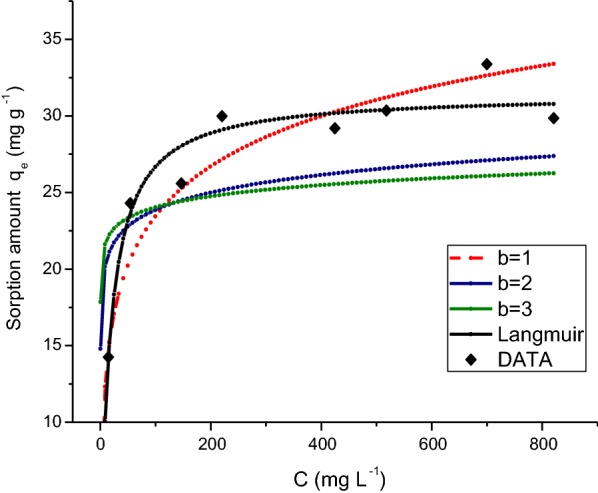
Illustration of the fit of the non-linear Langmuir model for adsorption of glyphosate onto ferrihydrite with NaCl 0.01 mol L^−1^ and effect of varying the parameter *b* in the Equation: *c *= *a* exp (*b S*) *S/*(*S*_max_−*S*), [31] where *c* is the observed concentration; *S* is the observed adsorption and *S*_max_ is the maximum adsorption. *b* had the values indicated

Barrow et al. [32] studied the specific adsorption of organic and inorganic phosphates by variable-charge oxides. They described a modification to the Langmuir equation, which is appropriate for sorption of inorganic phosphate and organic phosphate. This equation was applied to the glyphosate adsorption onto ferrihydrite. However, the results did not fit well in this model equation (Fig. 4). An explanation could be that this model was used in aluminium oxide and hydroxides, and in this work, glyphosate was adsorbed onto ferrihydrite that it is an iron oxide-hydroxide.

Using the Langmuir model, a higher maximum adsorption capacity (b) was obtained with a higher NaCl concentration (Tables 2 and 3), confirming the results in Fig. 2, where a higher glyphosate adsorption in the presence of a higher NaCl concentration was observed. However, an increase of NaCl concentration showed a decrease of the equilibrium constant (k) (Table 2), suggesting that the interaction of glyphosate-ferrihydrite is higher in 0.01 mol L^−1^ of NaCl. It is probably because of their higher surface area that ferrihydrites showed higher adsorption than goethite and hematite (Table 3). However, ferrihydrite (2-lines) showed a maximum adsorption capacity of 107 mg g^−1^ of glyphosate (Table 3). This value was twice that for 0.1 mol L^−1^ NaCl and three times that for 0.010 mol L^−1^ NaCl as determined in this work (Tables 2, 3). The value of pH used, the electrolyte and the different times of adsorption can be some of the factors that change the maximum adsorption capacity. However, these differences can also be explained by the degree of crystallinity of ferrihydrite. Gimsing and Borggaard [16] used a 2-lines ferrihydrite adsorbent with a surface area of 343 m^2^ g^−1^, while the ferrihydrite used in this work was the 6-lines type (Fig. 1) having a surface area of 234 m^2^ g^−1^ (Table 1). According to Michel et al. [56] there are no structural differences between ferrihydrite 2-lines and ferrihydrite 6-lines. The differences between them are due to particle size and the degree of crystallinity. Ferrihydrite 6-lines has a particle size from 5 to 7 nm and ferrihydrite 2-lines has a particle size from 2 to 4 nm. This difference is evidenced by the higher surface area of ferrihydrite 2-lines compared to ferrihydrite 6-lines (Table 3). This would explain the higher glyphosate adsorption capacity by ferrihydrite 2-lines.

### Adsorption kinetics

The curve of adsorption of glyphosate onto ferrihydrite showed that even after 180 min equilibrium was not reached (Additional file 3: Figure S3). This result is in accordance with Gimsing and Borggaard [16] who showed that even after six days the adsorption equilibrium of glyphosate onto ferrihydrite was not reached. However, the adsorption of glyphosate onto hematite and goethite reached equilibrium in a few hours [15, 16, 20]. Strauss et al. [57] have shown that the adsorption kinetics of phosphate on goethite depended on the crystallinity of the goethite. Since goethite and hematite have high crystallinity and ferrihydrite has low crystallinity, the crystallinity of these materials is probably playing an important role in the adsorption kinetics.

A pseudo-second-order model showed the best fit with the experimental results (Table 4). This is another indication that the glyphosate adsorption onto ferrihydrite occurs by inner sphere complexation [58, 59]. In addition, for this model, the q_e_ obtained (48.8 mg g^−1^) (Table 5) was close to the theoretical limit for adsorbed glyphosate (54.88 mg g^−1^) determined using the Langmuir isotherm model (Table 2). However, it can be seen in Table 4 that the correlation coefficients for the pseudo first order and intra-particle diffusion models were much lower than 1. This indicates that the rate-limiting step for the adsorption of this material is not intra-particle diffusion. This result is consistent with the parameters obtained in BET isotherm measurements indicating that the ferrihydrite is a microporous material with very small pores (Table 1). The entry of glyphosate into the small ferrihydrite pores is probably not possible. According to Barrow [60], an initial adsorption reaction is followed by a penetration of the adsorbed ions into the interior of the reacting particles. Thus, if intra-particle diffusion occurs, this mechanism is too slow for ferrihydrite and it cannot be observed at the studied period of time (180 min).

**Table 4:** Parameters obtained from the kinetic study of adsorption of glyphosate onto ferrihydrite

Model applied	Parameters	Values
Pseudo-first-order	k_1_	0.0023
	q_e_	0.61
	R^2^	0.083
Pseudo-second-order	k_2_	0.0032
	q_e_	48.8
	R^2^	0.991
Intra-particle diffusion	k_p_	0.390
	C	40.9
	R^2^	0.227

Experiments were carried out at temperature 307.6 K. Each value is a mean of three results. q_e_ is the glyphosate adsorption capacity for adsorbent (mg g^−1^) at equilibrium; k_1_ is the rate constant of pseudo-first-order kinetic model (min^−1^); k_2_ is the rate constant of pseudo-second-order kinetic model (g mg^−1^ min^−1^); k_p_ is the intra-particle diffusion rate constant (g mg^−1^ min^−0.5^); C is the intercept, which represent the thickness of the boundary layer

**Table 5:** Assignments of frequencies (cm^−1^) in FTIR spectra of ferrihydrite, glyphosate and glyphosate adsorbed onto ferrihydrite

FeHydr (cm^−1^)	GPS (cm^−1^)	Glyphosate adsorbed onto ferrihydrite (cm^−1^)				Tentative assignments
		pH 2.0	pH 4.0	pH 5.0	pH 6.0	
	1731^b^					C=O Stretching^3^
1635^a^						Adsorbed or lattice water^1, 2^
		1604^d^	1607^d^	1605^c^	1602^d^	C–O stretching^6, 8^
	1556^b^					NH_2_^+^ deformation^4^
		1534^d^	1528^d^	1530^c^	1530^d^	NH_2_^+^ deformation^6−8^
	1481^b^					NH_2_^+^ DEFORMATION^4^
1472^a^						Carbonate^1, 2^
		1444^d^	1445^d^	1452^c^	1450^d^	NH_2_^+^ deformation^7, 8^
	1432^b^					CH_2_ group^4^
	1420^b^					CO, OH group^4^
		1376^d^	1380^d^	1377^c^	1382^d^	C–O stretching^6^
1345^a^						Carbonate^1, 2^
	1334^b^					CH_2_ deformation^4^
		1314^d^	1318^d^	1316^c^	1320^d^	Carbon backbone coupled with C–O and P–O stretching^6^
		1270^d^	1268^d^	1269^c^	1268^d^	PO_3_H^−^ group (P=O)^4^
	1267^b^					PO_3_H^−^ group (P=O)^4^
	1242^b^					CH_2_ group^4^
	1220^b^					P–OH^4^
	1200^b^					CO, OH group^4^
		1119^a^	1123^a^	1122^a^	1121^a^	P–O stretching^6, 7^
	1088^b^					PO_3_Stretching^3, 4^
	1077^b^					P–O-stretching, PO_2_(OH-) group^5^
1076^a^						Carbonate^1, 2^
		1049^a^	1049^a^	1048^a^	1048^a^	CCNC skeletal vibration^7^
	1030^b^					CCNC skeletal vibration^4^
		987^a^	981^a^	983^a^	980^a^	P-OFe stretching^6^
	978^b^					Symmetric stretching P–O^3, 5^, CH_2_ group^4^
	909^b^					Stretching P–O^5^, CH_2_ deformation^4^, CCNC skeletal vibration^4^
896^a^						Carbonate^1, 2^
	864^b^					COH deformation^4^
	828^b^					POH deformation^4^

*FeHydr* ferrihydrite, *GPS* glyphosate

^1^Mazzetti and Thistlethwaite [36]; ^2^Ristić et al. [37]; ^3^Barja and Afonso [21]; ^4^Miano et al. [24]; ^5^Undabeytia et al. [23]; ^6^Sheals et al. [17]; ^7^Barja and Afonso [19]; ^8^Barja et al. [22]

**Fig. 5:**
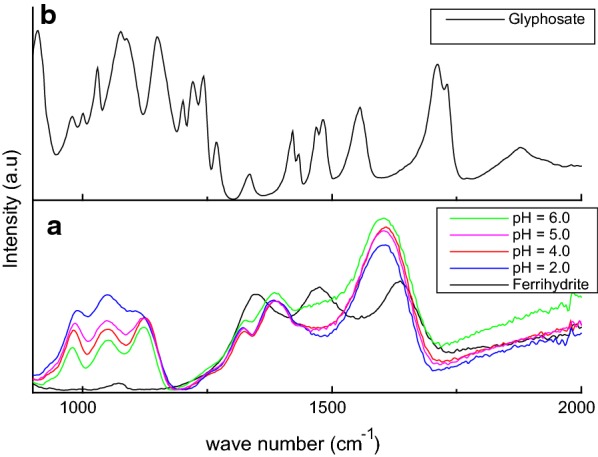
Infrared spectra of lyophilized samples **a** ferrihydrite and glyphosate adsorbed onto ferrihydrite at pH 6.0, 5.0, 4.0 and 2.0 and **b** glyphosate The adsorption of glyphosate onto ferrihydrite was performed in NaCl solution (0.10 mol L^−1^)

^a^ peaks from the Fig. 4a; ^b^peaks from the Fig. 4b, ^c^peaks from Fig. 5a; ^d^Figure not shown

### Desorption of glyphosate

Desorption of glyphosate was performed with two extractors, CaCl_2_ and Mehlich extractant. It should be noted that methodology recommends for desorption with CaCl_2_ the mass in g/volume in mL, 1.0:2.5 ratio, and for Mehlich the mass in g/volume in mL, 1.0:5.0 ratio [33, 34]. However, we performed both extractions with mass in g/volume in mL of 1.0:33.0 ratio, that it was much higher than recommended. We did this, because we want to test if glyphosate was strongly bonded to ferrihydrite. After three extractions using the CaCl_2_ extractor, only a small amount of desorbed glyphosate (< 3%) was observed. For the Mehlich extractor, after three extractions, the percentage extracted was approximately 16%. This occurs because a strong bond (covalent bond) forms between the glyphosate and ferrihydrite. Consequently, in soils with high amount of ferrihydrite glyphosate will not be easily desorbed under these conditions. Ferrihydrite is not easily identified in soils. However, for several reasons, in recent years there has been increased interest in identifying it. Thus, ferrihydrite has been identified in several soils around the world [8]. As pointed out by Borggaard and Gimsing [2], knowledge about the transport of glyphosate from land to water environments is very limited but it could occur in some soils. Thus, ferrihydrite in soils could play an important role on the preservation of water environments, since very little adsorbed glyphosate can be naturally extracted from it. For the adsorption of glyphosate onto goethite, using water or CaCl_2_ extractors and Mehlich extractor, the desorption of glyphosate can reach 60% and 73%, respectively [20].

### Infrared spectroscopy (FT-IR)

**Fig. 6:**
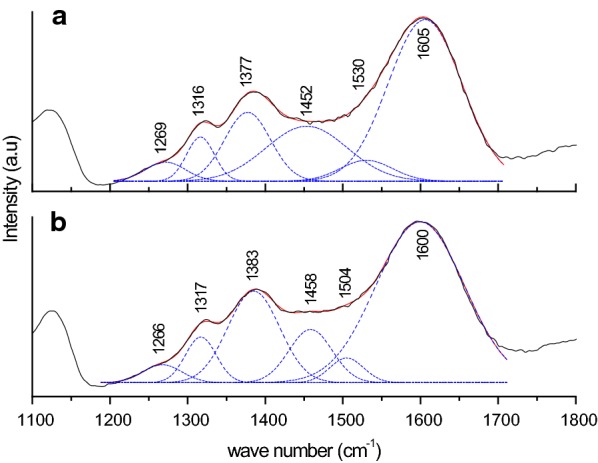
Deconvolution of the spectra glyphosate absorbed onto ferrihydrite at pH 5.0 and in presence of NaCl (**a**) 0.1 mol L^−1^ and (**b**) 0.01 mol L^−1^. The deconvolution of the bands of glyphosate adsorbed onto ferrihydrite were done in the range from 1189 to 1449 cm^−1^ for (**a**) and in the range from 1187 to 1458 cm^−1^ for (**b**). The best fittings were obtained with 6 bands (r^2^ = 0.999) for (**a**) and 6 bands (r^2^ = 0.998) for (**b**), in the Origin Program 8.0

In general, the spectrum of glyphosate as well as the spectra of glyphosate adsorbed onto ferrihydrite could be divided in two main regions: bands below 1300 cm^−1^ are due to the phosphate group of glyphosate and bands from 1800 to 1300 cm^−1^ are due to the amine and carboxyl groups of glyphosate (Figs. 5 and 6; Table 5).

Since only 16% of the glyphosate was desorbed from ferrihydrite by the Mehlich extractor, the glyphosate was strongly bonded to ferrihydrite. Thus, several bands of glyphosate shifted when it adsorbed onto ferrihydrite including: C–O stretching, NH_2_^+^ deformation, CCNC skeletal vibration and PO_3_H^−^ group (P=O) vibration (Figs. 5 and 6; Table 5). Glyphosate also withdrew carbonate from the ferrihydrite because the bands belonging to carbonate vanished (Table 5).

The band due to P–O stretching did not show a shift down with the increase in pH (Table 5). However, for the adsorption of glyphosate onto goethite, Scheals et al. [17] observed, with an increase in pH, a shift down of this band that was due to deprotonation of the amine group. The interaction of the amine group with the phosphate group caused this shifting [17]. Since our spectra did not show shifting of PO with pH, it is probable that the lack of shifting of NH_2_^+^ deformation could be due to an interaction with the iron in ferrihydrite (Table 5). Orcelli et al. [20] also showed that glyphosate interacts with goethite through phosphate and amine groups. However, Dideriksen and Stipp [18], using atomic force microscopy, observed that glyphosate interacts with goethite through the phosphate and carboxylic groups. In addition, the phosphate group interacts with the iron of ferrihydrite as shown by the stretching of the P-OFe band (Table 5).

### Environmental importance

It is important to note that, in the literature, there is a great lack of studies of the interaction of glyphosate with ferrihydrite. The main result of this work was that—even using three extractions with Mehlich-1 solution—only about 16% of the glyphosate was desorbed from ferrihydrite. Thus, in soils with a high content of ferrihydrite, glyphosate will probably not contaminate groundwaters or rivers easily, because it strongly interacts with this mineral. It should be also be pointed out that the adsorption of glyphosate by ferrihydrite is high when compared to other iron oxides (Table 3). Currently, due to saline irrigation water, soluble fertilizers, weathering of rocks by rain, and the capillary rise of saline groundwater and seawater, an increase in the number of areas with salt-affected soils has been observed [61]. However, at pH 4.0 and pH 6.0 (Fig. 2), an increase in salt content increased the adsorption of glyphosate onto ferrihydrite; for goethite this effect was observed at pH 7.0 [15]. Thus, even in areas with salt-affected soils, if the soils have a high content of ferrihydrite, glyphosate will probably not be washed out to groundwater or rivers under pH conditions typical of most soils. It should also be pointed out that it was determined that ferrihydrite showed a high affinity for sodium and chloride. At low concentrations of salt only chloride was adsorbed, however at high concentrations, because of the effect of ion pairs, both sodium and chloride were adsorbed. Thus, in soils with high concentrations of salts, ferrihydrite could also play an important role in decreasing the concentration of vegetation-affecting salt.

## Conclusions

Since, a low content of glyphosate (16%) was desorbed from ferrihydrite by Mehlich-1 extractor, most of glyphosate was strongly bonded to ferrihydrite as inner-sphere complex. Thus, in soils with high amount the ferrihydrite, glyphosate could not be easily washed out to groundwater or rivers. This result is in agreement to Langmuir isotherm model and pseudo second order model, since they assume the existence of only one adsorption site and chemisorption, respectively. Also, nonlinear Langmuir model and pseudo second order model showed similar result for the theoretical limit of adsorbed glyphosate onto ferrihydrite, 54.88 µg mg^−1^ and 48.8 µg mg^−1^, respectively. In addition, FT-IR spectroscopy showed that, the interaction between glyphosate and ferrihydrite was through phosphate and amine groups. EPR spectroscopy did not show dissolution of ferrihydrite under the experimental conditions used in this work. An increase of the pH caused a decreased in glyphosate adsorption. However, an increase in sodium chloride concentration augmented the adsorption of glyphosate onto ferrihydrite, even when the pH increased. Adsorption of glyphosate onto ferrihydrite decreased significantly surface area, pore volume and pH_pzc_ of ferrihydrite.

## Highlights

1. There is a great lack of studies on the interaction between glyphosate and ferrihydrite.
2. Soils with ferrihydrite, glyphosate will probably not contaminate groundwaters or rivers.
3. Adsorption of glyphosate by ferrihydrite is high when compared to other iron oxides.
4. Glyphosate will most likely not be washed out to groundwater or rivers in salt-affected soils.
5. Ferrihydrite could play a role in decreasing the concentration of vegetation-affecting salt.

## Additional files

**Additional file 1: Figure S1.** Molecular structures of glyphosate at different pHs. pk_1_ = 2.0; pk_2_ = 2.6;pk_3_ = 5.6;pk_4_ = 10.6 [62].

**Additional file 2: Figure S2.** EPR spectra of ferrihydrite (**———**) and glyphosate adsorbed onto ferrihydrite in the presence of 0.01 (**———**) and 0.10 (**———**) mol L^−1^ of NaCl.

**Additional file 3: Figure S3.** Adsorption kinetic of glyphosate onto ferrihydrite in a 0.10 mol L^−1^ NaCl solution and pH 5.0, at 307.6 K.

## Data Availability

All results of this article are available in the master thesis of Rodrigo C. Pereira.

## Abbreviations

glyphosate: *N*-(phosphonomethyl) glycine
EPR: electron paramagnetic resonance spectroscopy
pH_PZC_: pH at the point of zero charge
FT-IR: Fourier Transform infrared
DH: Dollimore and Heal
BJH: Barrett–Joyner–Halenda
BET: Brunauer, Emmett and Teller
