# Supplementary figures and images for: The effect of pH and ionic strength on the adsorption of glyphosate onto ferrihydrite
Source: Geochem Trans. 2019 May 24;20:3. doi: 10.1186/s12932-019-0063-1 (PMC6743134; doi:10.1186/s12932-019-0063-1)

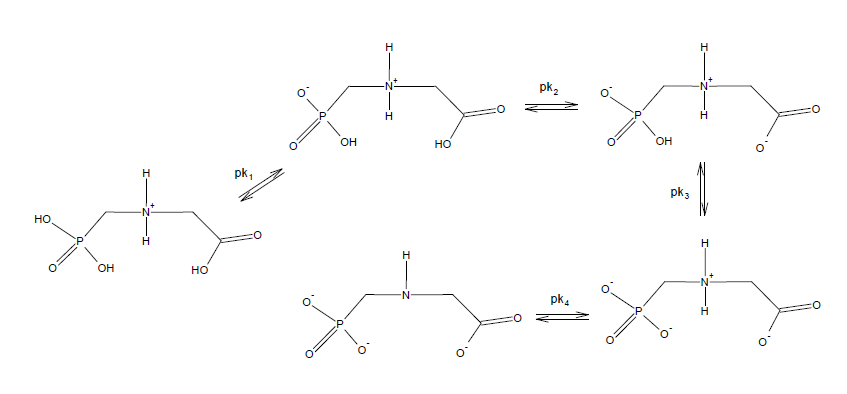


**Figure S1.** Molecular structures of glyphosate at different pHs. pk1=2.0; pk2 =2.6;pk3 =5.6;pk4 =10.6 [62].

Supplement: Supplementary file 1 — Additional file 1: Figure S1. Molecular structures of glyphosate at different pHs. pk1 = 2.0; pk2 = 2.6;pk3 = 5.6;pk4 = 10.6 [62]. [file 12932_2019_63_MOESM1_ESM.docx]
