# Supplementary material for: The effect of pH and ionic strength on the adsorption of glyphosate onto ferrihydrite
Source: Geochem Trans. 2019 May 24;20:3. doi: 10.1186/s12932-019-0063-1 (PMC6743134; doi:10.1186/s12932-019-0063-1)
Supplement: Supplementary file 2 — Additional file 2: Figure S2. EPR spectra of ferrihydrite (———) and glyphosate adsorbed onto ferrihydrite in the presence of 0.01 (———) and 0.10 (———) mol L−1 of NaCl. [file 12932_2019_63_MOESM2_ESM.docx]

**Figure S2.** EPR spectra of ferrihydrite (**⎯⎯⎯**) and glyphosate adsorbed onto ferrihydrite in the presence of 0.01 (**⎯⎯⎯**) and 0.10 (**⎯⎯⎯**) mol L^-1^ of NaCl.
