# Supplementary material for: The effect of pH and ionic strength on the adsorption of glyphosate onto ferrihydrite
Source: Geochem Trans. 2019 May 24;20:3. doi: 10.1186/s12932-019-0063-1 (PMC6743134; doi:10.1186/s12932-019-0063-1)
Supplement: Supplementary file 3 — Additional file 3: Figure S3. Adsorption kinetic of glyphosate onto ferrihydrite in a 0.10 mol L−1 NaCl solution and pH 5.0, at 307.6 K. [file 12932_2019_63_MOESM3_ESM.docx]

**Figure S3.** Adsorption kinetic of glyphosate onto ferrihydrite in a 0.10 mol L^-1^ NaCl solution and pH 5.0, at 307.6 K.
